# ZnO/Carbon Spheres with Excellent Regenerability for Post-Combustion CO_2_ Capture

**DOI:** 10.3390/ma14216478

**Published:** 2021-10-28

**Authors:** Iwona Pełech, Daniel Sibera, Piotr Staciwa, Ewelina Kusiak-Nejman, Joanna Kapica-Kozar, Agnieszka Wanag, Urszula Narkiewicz, Antoni W. Morawski

**Affiliations:** 1 Department of Inorganic Chemical Technology and Environment Engineering, Faculty of Chemical Technology and Engineering, West Pomeranian University of Technology in Szczecin, Pulaskiego 10, 70-322 Szczecin, Poland; Iwona.Pelech@zut.edu.pl (I.P.); daniel.sibera@zut.edu.pl (D.S.); piotr.staciwa@zut.edu.pl (P.S.); Ewelina.Kusiak@zut.edu.pl (E.K.-N.); joanna.kapica@zut.edu.pl (J.K.-K.); agnieszka.wanag@zut.edu.pl (A.W.); antoni.morawski@zut.edu.pl (A.W.M.); 2 Department of General Civil Engineering, Faculty of Civil and Environmental Engineering, West Pomeranian University of Technology in Szczecin, Piastow 50a, 70-311 Szczecin, Poland

**Keywords:** carbon spheres, carbon dioxide adsorption, microwave reactor, zinc oxide

## Abstract

This paper examines the synthesis of the ZnO/carbon spheres composites using resorcinol—formaldehyde resin as a carbon source and zinc nitrate as a zinc oxide source in a solvothermal reactor heated with microwaves. The influence of activation with potassium oxalate and modification with zinc nitrate on the physicochemical properties of the obtained materials and CO_2_ adsorption capacity was investigated. It was found that in the case of nonactivated material as well as activated materials, the presence of zinc oxide in the carbon matrix had no effect or slightly increased the values of CO_2_ adsorption capacity. Only for the material where the weight ratio of carbon:zinc was 2:1, the decrease of CO_2_ adsorption capacity was reported. Additionally, CO_2_ adsorption experiments on nonactivated carbon spheres and those activated with potassium oxalate with different amounts of zinc nitrate were carried out at 40 °C using thermobalance. The highest CO_2_ adsorption capacity at temperature 40 °C (2.08 mmol/g adsorbent) was achieved for the material after activation with potassium oxalate with the highest zinc nitrate content as ZnO precursor. Moreover, repeated adsorption/desorption cycle experiments revealed that the as-prepared carbon spheres were very good CO_2_ adsorbents, exhibiting excellent cyclic stability with a performance decay of less than 10% over up to 25 adsorption-desorption cycles.

## 1. Introduction

The intensification of the greenhouse effect in the last 200 years has contributed to the phenomenon of global warming. The cause of this phenomenon is the presence of greenhouse gases in the Earth’s atmosphere, such as water vapor, CO_2_, methane, N_2_O, and freons derived from natural processes or human activity. Carbon dioxide is one of the major anthropogenic factors influencing climate change. The increased CO_2_ concentration in the atmosphere is related to development of the energy industry, progressive urbanization and combustion of fossil fuels. For this reason, various materials have been tested as solid sorbents for CO_2_ capture: microporous zeolites [1], silica [2], activated carbons [3], ordered porous carbons [4], activated carbon fibers [3], graphene [5] and microporous carbon materials including carbon spheres. The increase of interest in these materials is due to their unique chemical and physical properties, such as high specific surface area, large porous volume, well-defined pore size distribution, chemical stability, low cost, the possibility for modification with heteroatoms, and high affinity for carbon dioxide [6,7,8,9,10,11,12]. In order to develop the surface area and improve the adsorption capacity of carbon materials, physical and chemical activation is one of the most frequently used methods. Physical activation is usually performed using CO_2_ or steam [13,14]. Potassium compounds such as KOH, K_2_CO_3_, K_2_C_2_O_4_ [9,15,16] or ZnCl_2_ [17] are often used for the chemical activation of carbon. For example, Ludwinowicz and Jaroniec obtained carbon spheres using potassium oxalate as an activator with a high surface area (2130 m^2^/g) and a high CO_2_ adsorption capacity equal 6.6 mmol/g at 0 °C [18]. Meng et al. investigated carbon materials activated with ZnCl_2_. The received materials had a specific surface area in the range of 539–1283 m^2^/g and exhibited CO_2_ adsorption capacity equal to 177 mg/g at 25 °C [19]. Wickramaratne and Jaroniec produced resin-derived carbon spheres and used potassium hydroxide as activating agent. The authors obtained carbon spheres with a specific surface area from 530 to 2400 m^2^/g. The highest achieved CO_2_ adsorption equalled 8.9 mmol/g at 0 °C [20].

The extraordinary properties of carbon materials can also be recombined with the properties of other elements, adding functional elements such as N [21], Fe [22], Ti [23] or Zn [24]. Thus, obtained carbon/metal oxide composites can be used in environmental applications such as gas sorption or contamination removal from water. For example, the addition of titanium oxide or zinc oxide to carbon material allows receiving carbon/metal oxide composites with photocatalytic properties.

It is known that a homogeneous distribution of metal oxide in the carbon matrix or the high values of CO_2_ adsorption capacity depend on the selected precursors and preparation method. To obtain micro-/mesoporous carbon, phenol or resorcinol resins are usually applied [25,26], and the Stöber method has been one of the most employed techniques [9,18,27]. The disadvantage of this method is the very long processing time which can be shortened up to several minutes using microwave radiation [28].

In our previous studies, we successfully employed a microwave reactor to obtain highly microporous carbon materials [7,28,29]. Spherical carbon materials about the specific surface area in the range from 439 to 1614 m^2^/g and characterized by the ability to perform CO_2_ adsorption at 0 °C and from 3.51 to 6.21 mmol/g have been received. Additionally, the materials had good stability over repetitive adsorption-desorption multicyclic analysis [7]. Adsorption of CO_2_ and methane was also investigated using the carbon spheres/TiO_2_ composites obtained in the microwave reactor [23]. The processes were conducted under ambient pressure and temperatures of 40, 60, and 80 °C. Values of carbon dioxide uptake equalled 3.94 mmol CO_2_/g at 40 °C and under 4 MPa. 

Solvothermal microwave reactors have also been used for the preparation of zinc oxide [30,31]. Wojnarowicz et al. [32] described the effect of water concentration in a reaction carried out in a microwave-assisted solvothermal reactor on the ZnO particle size. Depending on the synthesis parameters, ZnO particles had a size from 59 to 120 nm. 

The above examples allow for the conclusion that a solvothermal reactor heated with microwaves can be successfully used for the production of carbon materials, and also to obtain carbon materials doped with metals or metal oxides. This paper presents the results of the synthesis of microporous carbon spheres doped with zinc oxide in a microwave reactor. The resorcinol-formaldehyde resin was used as a carbon precursor. Zinc nitrate was applied as a precursor of zinc oxide. The aim of the described research was to investigate the effect of ZnO doping on morphology, microporous structure, and CO_2_ adsorption efficiency.

## 2. Materials and Methods

In order to obtain carbon spheres, 0.6 g of resorcinol was dissolved in an aqueous alcohol solution composed of 60 mL distilled water and 24 mL of ethanol. In the case of activated samples, an appropriate quantity of potassium oxalate (K_2_C_2_O_4_·1H_2_O) was added, and the mixture was stirred until the potassium oxalate was completely dissolved (the weight ratio of potassium:carbon was 7:1). Next, zinc nitrate (Zn(NO_3_)_2_·6H_2_O) was added, and the whole mixture was stirred until complete dissolution (the weight ratios of carbon:zinc was 10:1, 5:1 and 2:1). In order to adjust pH~9, ammonium hydroxide (25 wt.%) was dropped in to the solution. Finally, 0.9 mL of formaldehyde (37 wt.%) was added, and the whole mixture was mixed using a magnetic stirrer at ambient conditions to facilitate the polycondensation reaction. After 24 h, the mixture was transferred into an ERTEC MAGNUM II solvothermal reactor heated with microwaves. The processes were conducted under 2 MPa and the reaction lasted only 15 min. After treatment in the reactor, the samples were dried for 48 h at 80 °C and then carbonized in a high-temperature furnace (HST 12/400 Carbolite) under an argon atmosphere at a temperature rising from 20 to 350 °C with a heating rate of 1 °C/min and a holding time 2 h, and from 350 °C to 700 °C with a heating rate of 1 °C/min, respectively. The carbonization temperature (700 °C) was chosen based on our previous research. When a temperature of 700 °C was reached, carbonization continued for 2 h. Afterwards, the samples were cooled to room temperature under an argon atmosphere. The final products were washed with distilled water and dried for 48 h at 80 °C under atmosphere.

The received materials were denoted as RF for samples without the addition of potassium oxalate, or RF 7/1 with the addition of potassium oxalate. For example, RF7/1 + Zn10/1 refers to the activated sample modified using zinc nitrate (the weight ratio of carbon:zinc was 10 to 1).

The morphology of the samples was investigated using an SU8020 ultra-high resolution field emission scanning electron microscope (Hitachi Ltd., Chiyoda, Tokyo, Japan). The phase composition was investigated with X-ray diffraction using Cu Kα radiation (λCu Kα = 0.1540 nm) on an Empyrean panalytical. Phase identification was performed using HighScore+ and the ICDD PDF-4+ 2015 database. 

The FT-IR spectra of tested materials were recorded using a Thermo Scientific Nicolet 380 spectrometer (Thermo Fisher Scientific Inc., Waltham, MA, USA). The measurement of the spectra was performed in the region of 4000–400 cm^−1^ utilizing KBr (Merck KGaA, Darmstadt, Germany) pellets. Before pellet preparation, the KBr powder was sifted through a sieve of sieve size of 0.071 mm, and then dried at 110 °C for 24 h. In order to prepare the pellets, approx. 0.2 mg of the sample was mixed into 500 mg of KBr powder and then put into a pellet-forming die. Before that, the sample was outgassed in order to eliminate air and moisture from the KBr powder. The homogenized mixture of a KBr and tested sample was pressed into a transparent, colorless pellet using a 10-ton hydraulic press. For better visualization, all spectra were multiplied by 8. 

Thermogravimetric measurements were conducted to study the thermal stability of the tested sorbents within the temperature range of 25–950 °C. Thermal analysis was carried out to obtain data on the decomposition processes, thermal stability and temperature of phase transformations of the prepared materials. The thermal analysis was conducted using an STA 449 C thermobalance (Netzsch Company, Selb, Germany). The samples (ca. 10 mg) were placed in the TGA sample pan and heated from ambient temperature to 950 °C at a heating rate of 10 °C/min under airflow of 30 cm^3^/min.

Characterization of porosity was performed using N_2_ adsorption/desorption on a QUADRASORB evo^TM^ gas sorption automatic system (Quantachrome Instruments) at −196 °C. The Brunauer–Emmett–Teller (BET) equation was used to determine the surface areas (S_BET_), and S_BET_ was determined in the relative pressure range of 0.05–0.2. The total pore volume, TPV, was calculated from the volume of nitrogen held at the highest relative pressure (p/p_0_ = 0.99). 

The volume of micropores V_m_ with dimensions smaller than 2 nm was calculated as a result of integrating the pore volume distribution function using the DFT method; mesopore volume V_meso_ with dimensions from 2 to 50 nm was calculated from the difference of the total pore volume TPV and the volume of micropores V_m_. 

Before each adsorption experiment, samples were outgassed at 250 °C under a vacuum of 1 × 10^−5^ mbar for 12 h using a MasterPrep multi-zone flow/vacuum degasser from Quantachrome Instruments (Boynton Beach, FL, USA) to remove adsorbed species that could intervene in the adsorption processes. Carbon dioxide adsorption isotherms at 0 °C and 25 °C were measured using the same Quadrasorb™ automatic system (Quantachrome Instruments) in the pressure range between 0.01 and 0.98 bar. The pore size distribution (PSD) of the samples was calculated from the CO_2_ sorption isotherms at 0 °C using the NLDFT model. The volume of the ultramicropores V_s_ with dimensions smaller than 1.0 nm (< 1 nm) was determined from the CO_2_ adsorption isotherm at 0°C and calculated as a result of integrating the pore volume distribution function using the NLDFT method. 

CO_2_ adsorption-desorption measurements were additionally carried out using a Netzsch STA 449 C thermobalance. For this purpose, a sample of about 10 mg was put into the TGA sample crucible and then the temperature was raised to 105 °C at a rate of 5 °C/min under a 30 cm^3^/min N_2_ atmosphere and maintained at 105°C for 60 min. The purpose of this procedure was to remove some water from the samples. After 60 min, the sample was cooled to adsorption temperature (40 °C) and stabilized under these conditions for 120 min. After this time, the N_2_ was switched to pure CO_2_ (99.99%) under 30 cm^3^/min at a rate of 5 °C/min, and the carbon dioxide adsorption process began. After completing adsorption, the gas was switched back to N_2_ and heated to 105 °C for 60 min at a heating rate of 5 °C/min in order to desorb carbon dioxide. 

The adsorption–desorption cycle was repeated several times. The CO_2_ sorption capacity (mmol CO_2_/g of sorbent) of the tested samples was computed according to the weight change of the sample after each of the runs.

## 3. Results and Discussion

**Figure 1:**
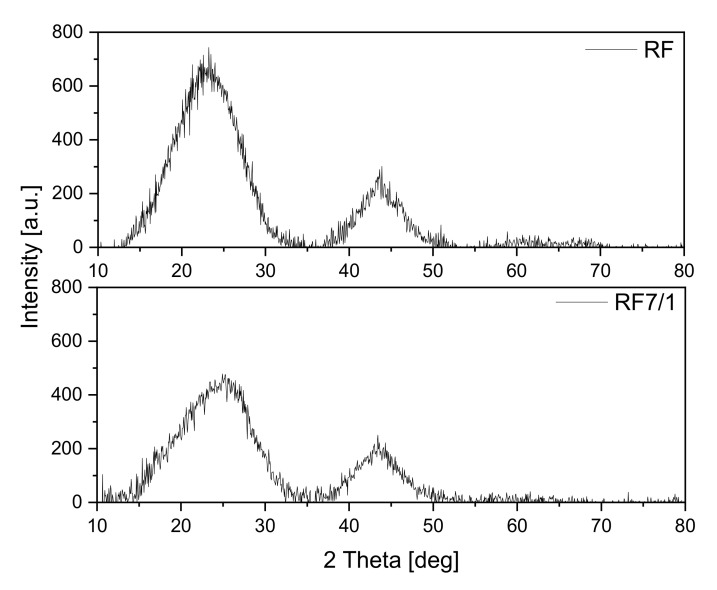
Diffraction patterns of the reference materials.

The obtained materials were investigated using the X-ray diffraction method, and the diffraction patterns are shown in Figure 1. The reference sample exhibited two diffraction peaks: the first at around 23° and the second at around 43°. Both can be assigned to carbon [33]: the first to the stacking carbon layer structure (002) and the second to the ordered graphitic carbon structure (100). In both cases, the peaks are broadened, which suggests a low degree of graphitization and the possible presence of amorphous carbon [34,35]. Additionally, in the case of the activated material, the peak width at 23° increases, and the maximum peak shifts towards the angle of 25° in comparison with a non-activated sample. These results indicate that the degree of carbon graphitization may decrease in the activation process.

**Figure 2:**
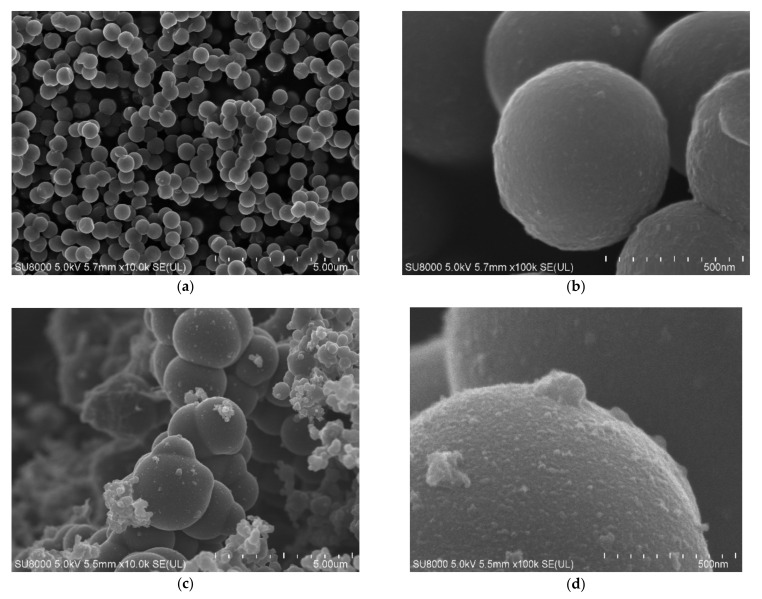
SEM images of the non-activated (**a**,**b**) and activated (**c**,**d**) materials.

The obtained materials were examined using scanning electron microscopy. In Figure 2, SEM images of the reference samples (non-activated RF and activated RF7/1 samples) are shown. For the non-activated sample, carbon spheres with regular shape, smooth surface, no inclusions, no impurities and with a mean diameter of about 600 nm were observed (Figure 2a,b). For the activated sample, a nonhomogeneous material with respect to the size of the carbon spheres was noticed (Figure 2c,d). Larger spheres with diameters up to 2000 nm appeared. More impurities and inclusions were observed on the surfaces of the carbon spheres. Furthermore, the merging of carbon spheres was noticed. According to [36], the presence of oxygen groups on the surface of carbon with CO_2_ at high temperatures may favor the phenomenon of joining single spheres together.

**Figure 3:**
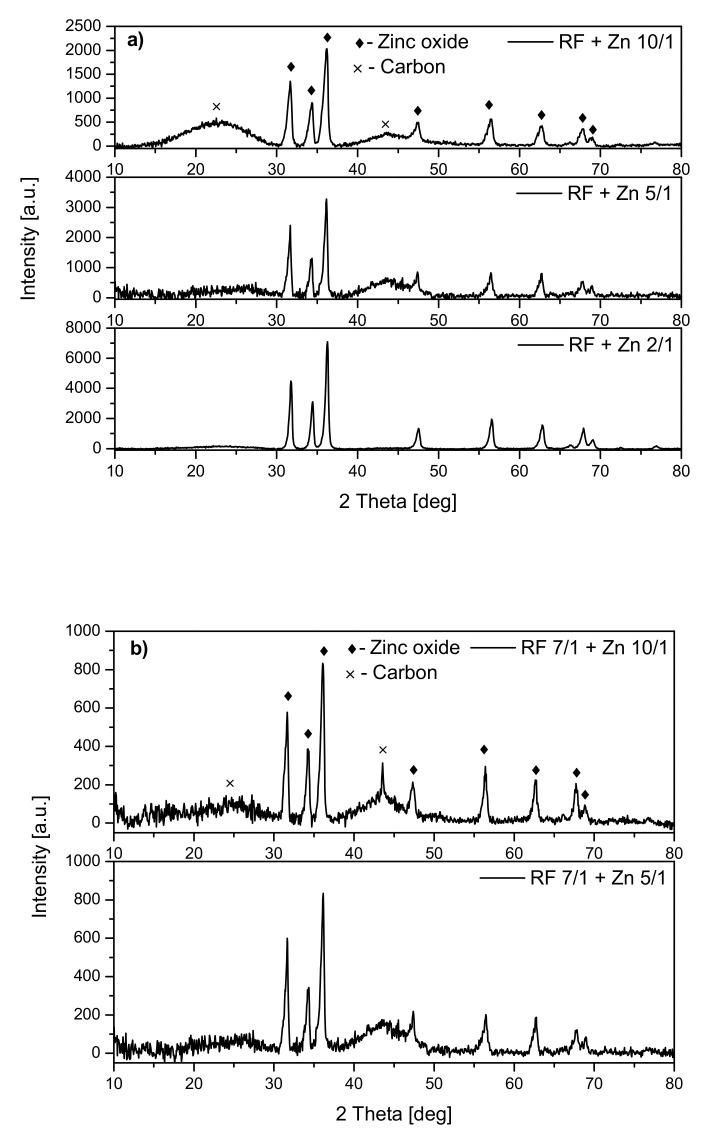
Diffraction patterns of non-activated (**a**) and activated (**b**) carbon materials modified with Zn(NO_3_)_2_ as ZnO precursor.

Analysis of the diffraction patterns of the samples modified with zinc nitrate (Figure 3) showed that the phase composition of all materials was the same. For all the samples, the peaks corresponding to zinc oxide were observed regardless of whether the material was activated (Figure 3b) or not (Figure 3a). Additionally, the peaks corresponding to carbon at around 23° and 43° were identified. No other crystalline phases were identified.

For the nonactivated samples, the increase of the ZnO peak intensity together with the increase of the weight ratio of carbon:zinc from 10:1 to 2:1 was noticed, which can confirm the presence of a higher amount of ZnO in the RF + Zn2/1 than RF + Zn10/1 material. 

**Figure 4:**
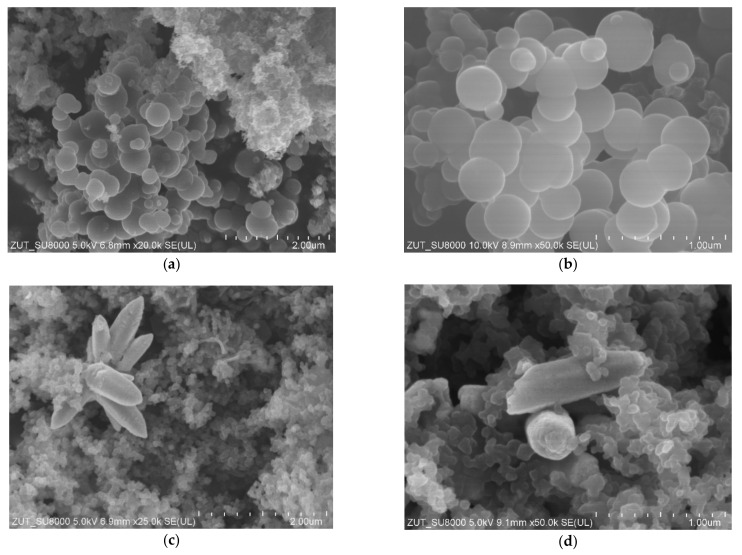
SEM images of non-activated RF + Zn10/1 (**a**,**b**) and RF + Zn2/1 (**c**,**d**) materials modified with Zn(NO_3_)_2_ as ZnO precursor.

In Figure 4, SEM images of non-activated carbon spheres modified with zinc nitrate are presented. In the sample with the 10:1 carbon:zinc weight ratio (Figure 4a,b) carbon spheres in diameters about 600 nm were visible, as in the case of the reference material (Figure 2a,b). Probably the addition of zinc nitrate as a ZnO precursor during the synthesis disturbed the growth of carbon spheres, resulting in a deterioration of material homogeneity, and smaller spherical structures appeared as well. The increase of the zinc oxide content in the sample (Figure 4c,d) led to the destruction of the spherical structures. Simultaneously in the RF + Zn2/1 sample, well-crystallized tubular and flower-like ZnO structures were clearly visible. Similar ZnO structures were synthesized by the precipitation method from zinc nitrate and ammonium hydroxide [37] or the sol-gel method using zinc acetate dihydrate and an ammonia solution [38].

**Figure 5:**
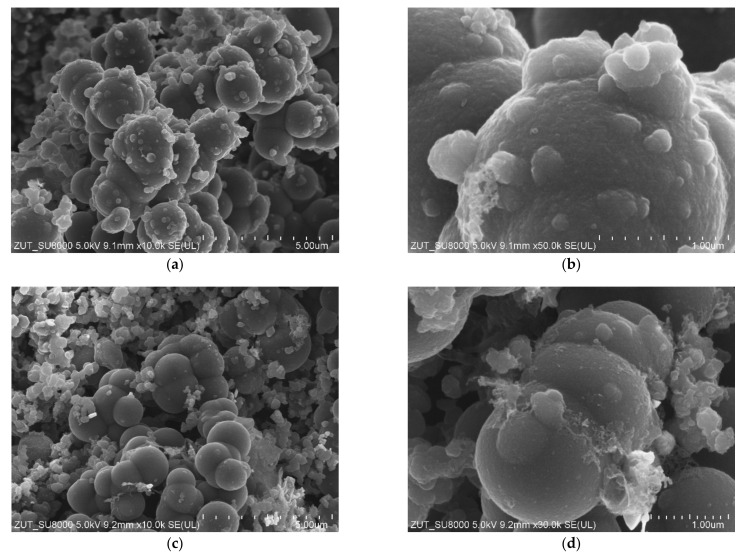
SEM images of activated RF7/1 + Zn10/1 (**a**,**b**) and RF7/1 + Zn5/1 (**c**,**d**) materials modified with Zn(NO_3_)_2_ as ZnO precursor.

In Figure 5, the images of activated carbon spheres modified with zinc nitrate are presented. Regardless of the amount of zinc oxide in the samples (RF7/1 + Zn10/1: Figure 5a,b and RF7/1 + Zn5/1: Figure 5c,d), carbon spheres with large diameters up to 2000 nm were observed. In comparison with the reference material, more inclusions and carbon impurities were noticed. The merging of carbon spheres was also visible. The influence of the activation process using potassium salt on the morphology and homogeneity of the obtained carbon spheres has been more widely discussed in our previous works [7,28,29]. Other authors also noticed that the activation of carbon spheres with potassium salts resulted in obtaining carbon spheres of larger diameters and irregular shapes [18,39]. So it seems that the major factor influencing the spherical morphology is potassium oxalate and not zinc oxide.

**Figure 6:**
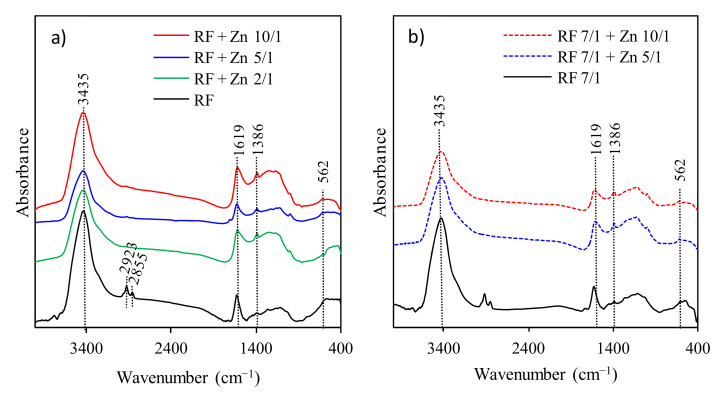

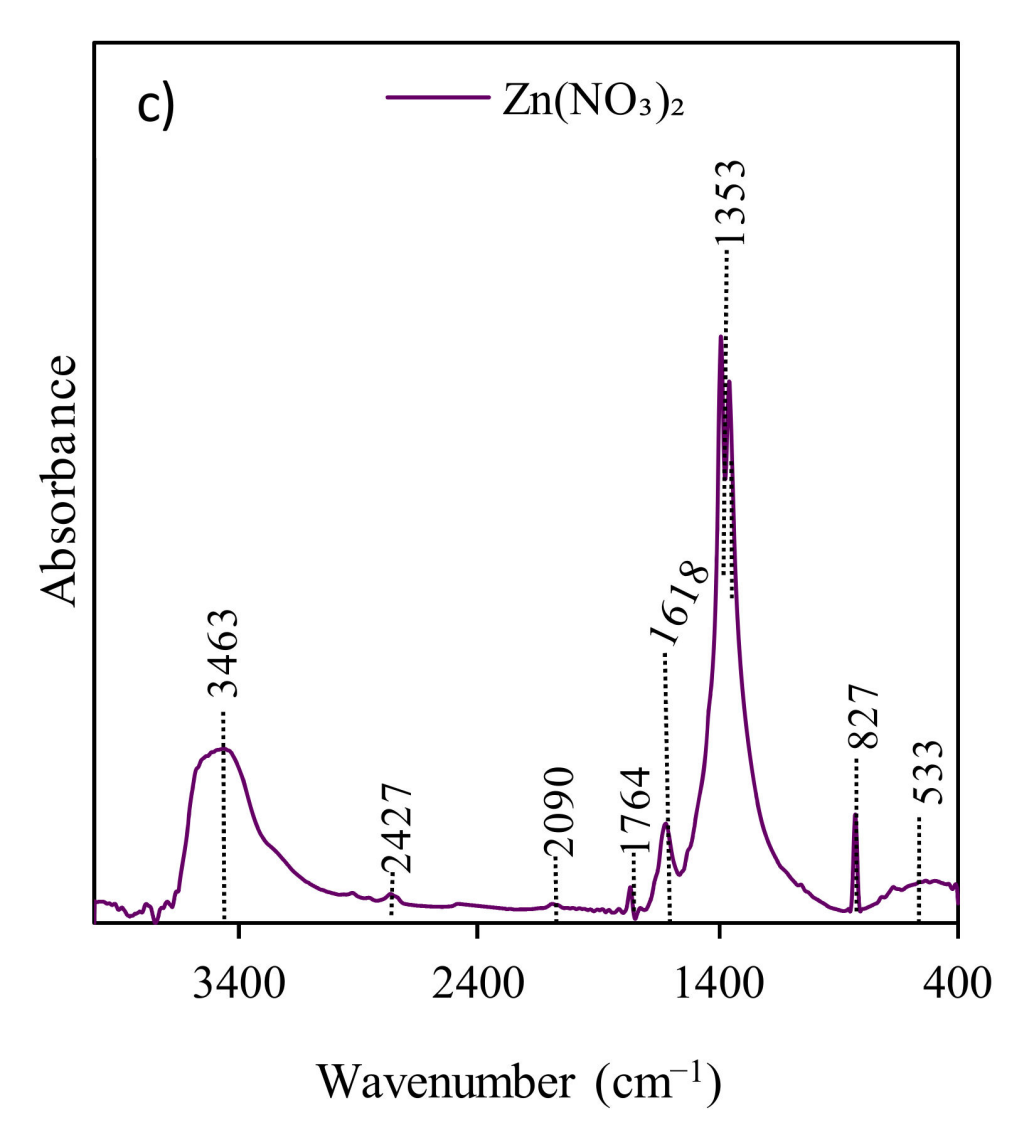
FT-IR spectra of carbon spheres modified with Zn(NO_3_)_2_ **(a)**; carbon spheres modified with Zn(NO_3_)_2_ and activated with potassium oxalate (**b**); reference spectra of Zn(NO_3_)_2_ salt (**c**).

The FT-IR spectra presented in Figure 6 were used to identify the presence of functional groups in all obtained RF samples.

The spectra of RF materials with and without the addition of potassium oxalate are similar. The broad absorption band at 3440 cm^−1^ is attributed to the stretching vibration of the –OH groups located on the surface of materials [17]. Generally, the bands at 2800–3000 cm^−1^ correspond to aliphatic –CH stretching [40]. The bands located at 2855 and 2923 cm^−1^ indicate the presence of the C–H symmetric and asymmetric stretching vibrations, respectively [17]. The bands at 1627 and 1384 cm^−1^ are related to the skeleton vibration of aromatic C=C, and the O–H in-plane deformations [41]. The broad medium-strong bands between 1100 and 1250 cm^−1^ correspond to the C–O stretching and C–OH bending vibrations, indicating a presence of residual hydroxyl groups constituting the hydrophilic surface [17,42]. All samples after modification with zinc nitrate generally presented similar patterns to RF materials. No changes after modification were observed. This phenomenon is related to a very similar procedure of sample preparation. 

**Figure 7:**
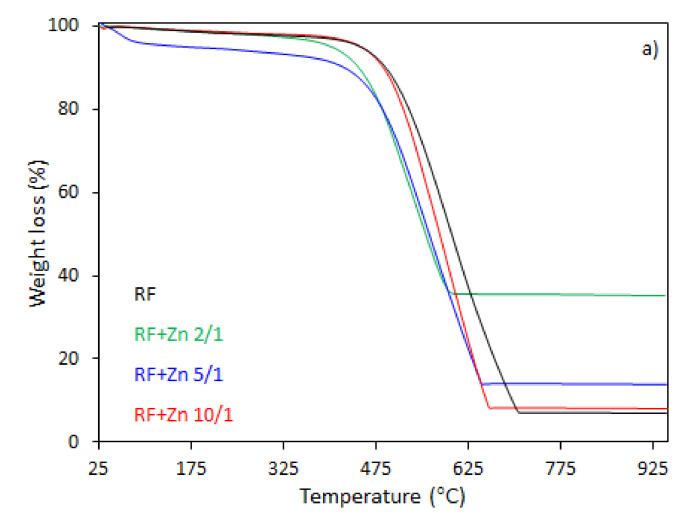

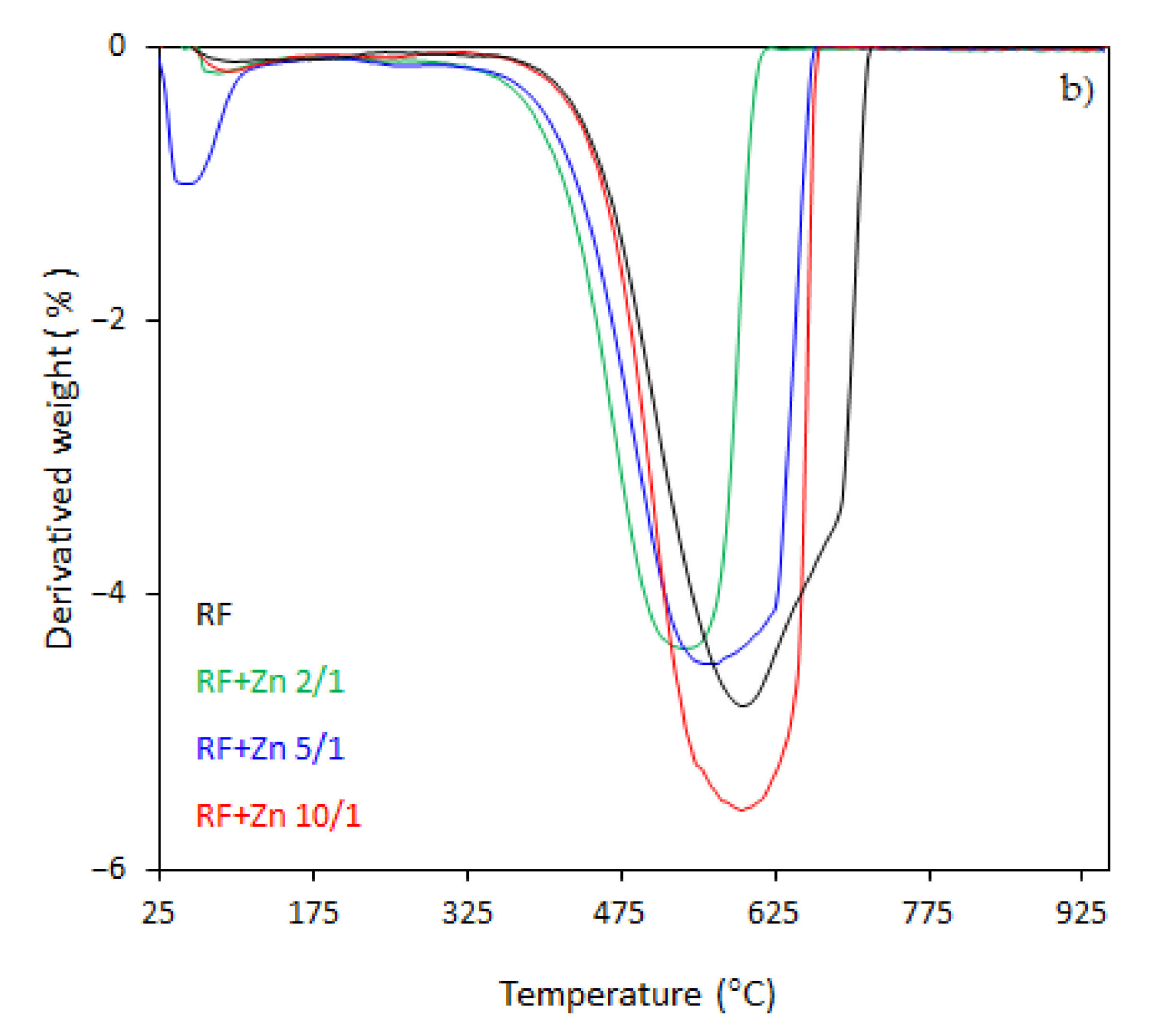
TG (**a**) and DTG (**b**) curves of the unmodified RF and carbon spheres modified with different amounts of zinc nitrate.

**Figure 8:**
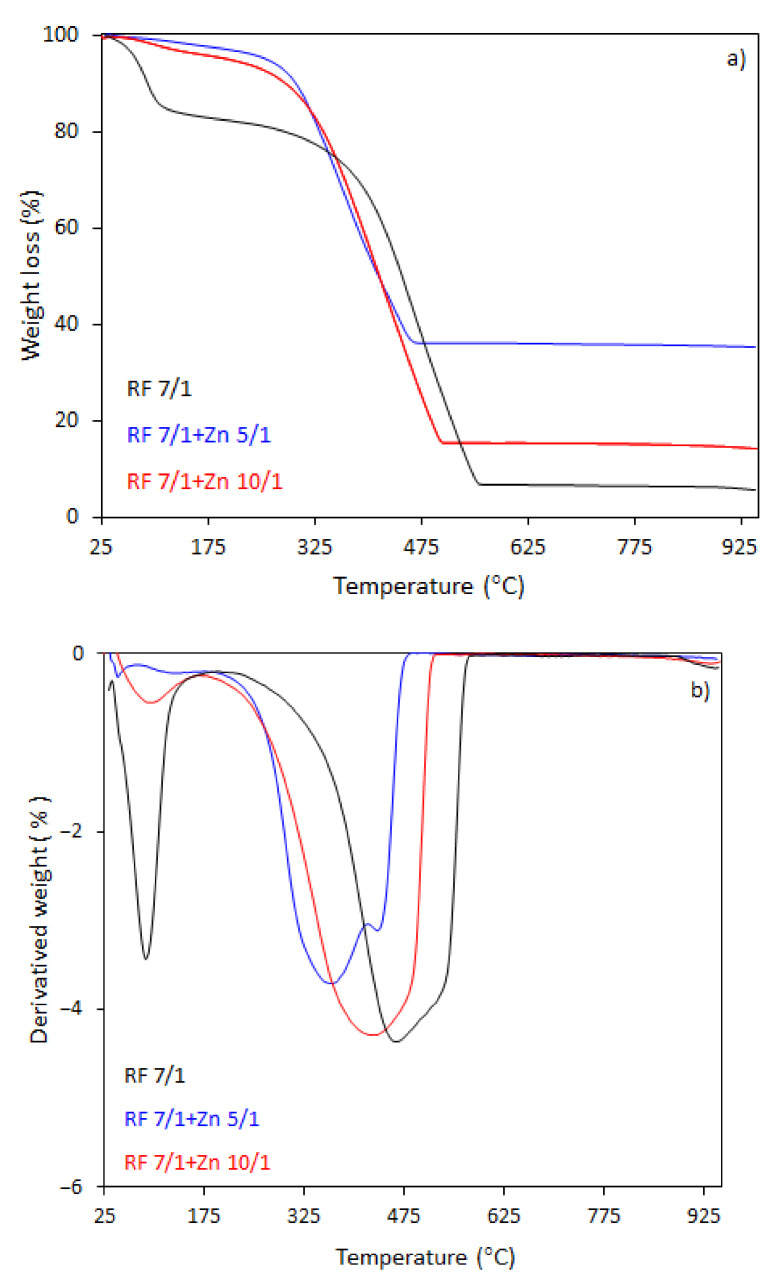
TG (**a**) and DTG (**b**) curves of the activated RF and carbon spheres modified with different amounts of zinc nitrate after activation with potassium oxalate.

The thermal behavior of the carbon spheres modified with different amounts of zinc nitrate, either nonactivated (Figure 7), or activated with potassium oxalate (Figure 8), was determined using thermogravimetry (TG) and differential thermogravimetry (DTG) analysis (Figure 7a,b and Figure 8a,b, respectively). 

In the case of the unmodified sample (RF), the total weight loss of about 93% was observed between 330 and 730 °C, corresponding to the differential thermogravimetric (DTG) profile with a maximum at 600 °C, which could be attributed to the decomposition of the RF resin network, forming a carbon material of porous nature. The mass loss after the modification of samples began at 25–150 °C. This may be assigned to the dehydration of the water or crystallization [43]. It should be noticed that with the increased degree of coalification of the initial material, the temperature shifted towards lower values. The samples modified with different amount of zinc nitrate exhibited less weight loss within the range of temperature of 328–670 °C and 321–650 °C, corresponding to the DTG profile centered at 600 and 570 °C for the samples RF + Zn 10/1 and RF + Zn 5/1, respectively. The total weight loss for the sample RF + Zn 10/1 was 92%, which decreased to ca. 86% for the RF + Zn 5/1 sample, whereas the DTG profile for the RF + Zn 2/1 (sample synthesized at a highest zinc nitrate ratio) showed a beginning of the thermal decomposition process at 269 °C and ending at 620 °C, reaching the maximum value at 530 °C, while the total weight loss was 65%. According to the thermogravimetric (TG) study, the weight loss decreased with the increasing ZnO content in the composites (confirmed by XRD). 

The TG-DTG curves of samples activated with potassium oxalate are shown in Figure 8a,b. The activated samples had a lower initial combustion temperature than the non-activated samples. The total weight loss for the sample RF7/1 was 94%, which was accompanied by a broad mass loss peak in the DTG profile. Decomposition occurred in the range of 160–570 °C, reaching the maximum rate of decomposition at 460 °C (DTG profile). The initial decomposition temperature was about 180 °C lower then initial decomposition temperature for RF sample. The total weight loss for the sample RF7/1 + Zn 10/1 was 86%, whereas for the samples RF7/1 + Zn 5/1 the losses reached 65%. The residuals were about 6.5% and 25.6%, respectively, smaller than to total weight losses for the non-activated carbon spheres.

As we can clearly see from Figure 8a,b, the ZnO modification of activated carbon spheres results in the decrease of the thermal stability of carbon spheres. The effect of potassium oxalate as an activator agent has been explained in our previous work [29,44]. Briefly, activation with K_2_C_2_O_4_ results in the formation of additional channels with pores. Therefore, during heating, oxygen has easier access to a larger surface of the material. Moreover, the application of potassium oxalate as an activator resulted in higher hydrophilicity of the material because the negatively charged side of the water molecules is attracted to the positively charged potassium ions, which caused the combustion of materials at lower temperatures compared to those prepared without addition potassium oxalate. Moreover, in the case of the material with the highest precursor (Zn(NO_3_)_2_·6H_2_O as a ZnO source) content, the presence of two peaks in the DTG curve indicates that decomposition is a double-stage process. Such changes were not observed in the case of the material before the activation process. The first weight loss at the temperature range 180–417 °C with a decomposition peak at 360 °C in the DTG curve and the total weight loss 48% may be attributed to the partial dehydratation of Zn(NO_3_)_2_·6H_2_O, first to zinc nitrate(V) tetrahydrate and then to dihydrate. The second weight loss at the temperature range 417–490 °C, reaching the maximum rate of decomposition at 440 °C with a total weight loss of 13% corresponds to the decomposition of the intermediate to ZnO [45,46] or the presence of the other forms of carbon (e.g., amorphous or graphitic carbon)

**Figure 9:**
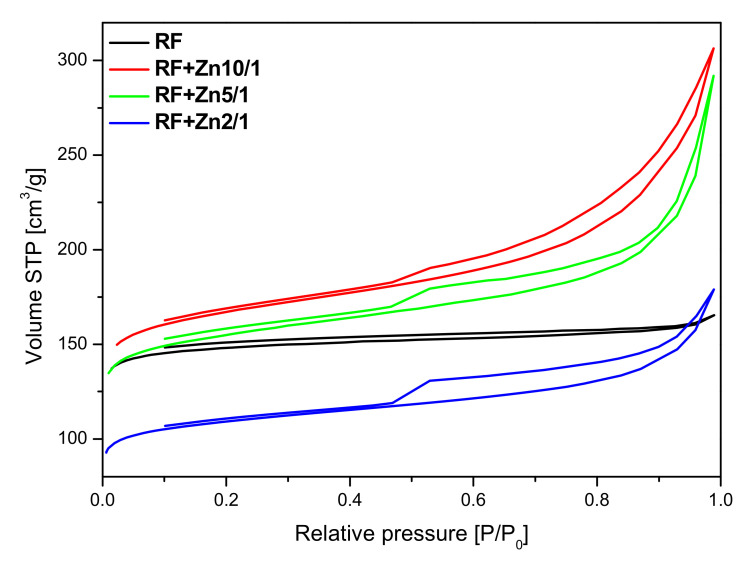
Nitrogen adsorption and desorption isotherms of the non-activated sample and the non-activated sample modified with zinc nitrate.

The nitrogen adsorption isotherms measured on the non-activated materials modified with zinc nitrate are given in Figure 9. For the unmodified RF sample, an isotherm of type I according to the IUPAC classification, characteristic for the microporous materials was obtained. Modification of the carbon material with Zn(NO_3_)_2_ resulted in a decrease in the specific surface area. Furthermore, the nitrogen isotherms obtained for these materials are of type II, characteristic of macroporous materials with H4 hysteresis loops. It can be concluded from the nitrogen adsorption isotherms that, with the increase of the Zn content, modified samples express higher content of macropores.

The presence of adsorption/desorption hysteresis loops in the samples modified with zinc nitrate may indicate on a very high length to width ratio of the pores or the so-called bottle shape of the pores—the pores are wider in the depth of the grain of the material than at the edge. In our materials modified with zinc nitrate, we observed the H4 type of hysteresis loop, which is often observed in micro-mesoporous carbon materials.

**Figure 10:**
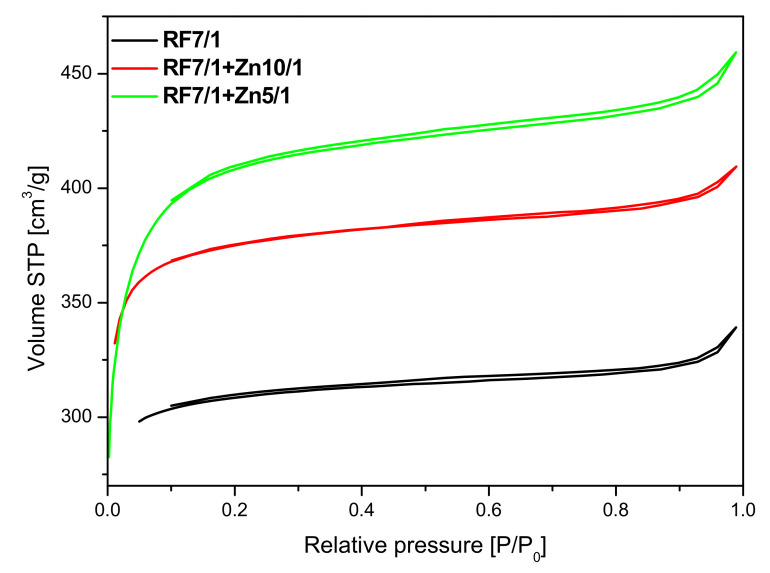
Nitrogen adsorption and desorption isotherms of the activated sample and activated samples modified with zinc nitrate.

As can be seen in Figure 10, the unmodified activated carbon material RF 7/1 expressed the type I nitrogen isotherm, characteristic for microporous materials. A similar shape of the isotherms was obtained for the activated materials modified with Zn(NO_3_)_2_^.^ 6H_2_O. The application of zinc nitrate hexahydrate as a ZnO source also resulted in an increase in the specific surface area value. Moreover, the microporosity of the samples was preserved.

**Figure 11:**
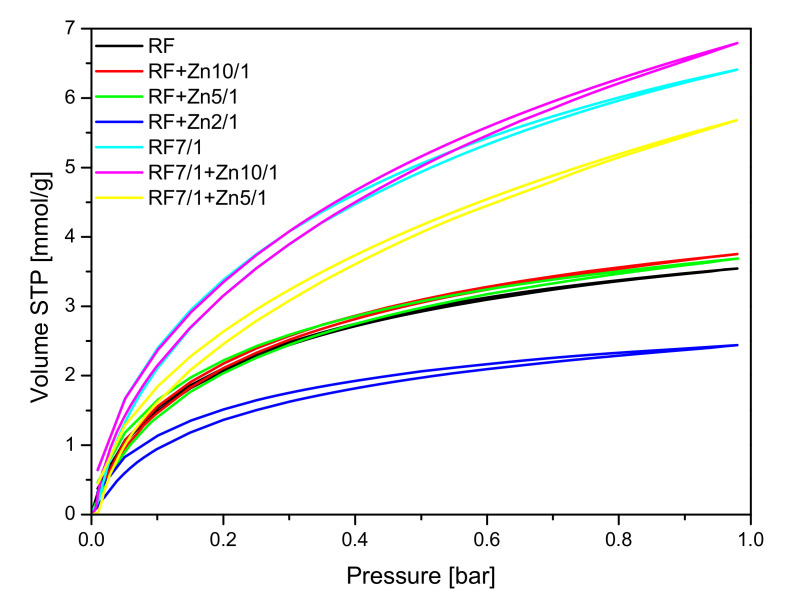
CO_2_ sorption isotherms at 0 °C for the reference materials and samples modified with zinc nitrate.

The parameters characterizing the porous structure of the tested materials were determined on the basis of low-temperature (−196 °C) nitrogen adsorption isotherms (Figure 9 and Figure 10) and on the basis of CO_2_ adsorption at 0 °C (Figure 11). On the basis of the nitrogen adsorption isotherms, the parameters characterizing the porous structure: S_BET_ specific surface area, total pore volume TPV, volume of micropores V_m_ (< 2 nm), and mesopore volume V_meso_ of the tested materials were determined and listed in Table 1.

The surface area of the non-activated reference material RF equalled 455 m^2^/g. The highest value of the surface area was achieved for the non-activated sample modified using zinc nitrate RF + Zn10/1 and equaled to 524 m^2^/g. The increase of the amount of the precursor in the material reduced the values of the surface area down to 487 m^2^/g and 342 m^2^/g for the RF + Zn5/1 and RF + Zn2/1 materials, respectively. 

As expected, activation of carbon spheres using potassium oxalate resulted in the development of a specific surface area compared with the non-activated materials. It was noticed that the highest surface area was obtained for the sample modified using zinc nitrate RF7/1 + Zn5/1 and equaled to 1268 m^2^/g. It was also clearly visible that the values of surface area correlated well with the values of the total pore volume. It means that together with the increase of the values of surface area the values of total pore volume increased. It could be expected that, for the material with the highest value of surface area and with the highest value of total pore volume, the highest values of CO_2_ adsorption should be obtained. 

CO_2_ sorption isotherms at 0 °C are given in Figure 11, and the values of CO_2_ adsorption at 0 °C and 25 °C aand the values of the volume of ultramicropores with dimensions smaller than 1 nm calculated based on CO_2_ adsorption isotherms at 0 °C are presented in Table 1. 

The collected results showed that modification of the non-activated carbon material with Zn(NO_3_)_2_ where the carbon-zinc ratio was 10/1 resulted in an increase in the specific surface area and in the total pore volume, but only in a slight increase of CO_2_ adsorption values. For the reference material (RF) CO_2_ adsorption equaled to 3.25 mmol/g at 0 °C and 2.43 mmol/g at 25 °C, but for the modified material (RF + Zn10/1), the 3.75 mmol/g at 0 °C and 2.13 mmol/g at 25 °C values of CO_2_ adsorption were reached. For the modified material RF + Zn5/1, a similar dependence was observed. It is worth noting that the increase of total pore volume for both materials RF + Zn10/1 and RF + Zn5/1 was associated only with the increase of the volume of mesopores in the samples. The volumes of ultramicropores and micropores remained at a similar level, as with the reference sample. Therefore similar values of CO_2_ adsorption were obtained for both samples. When the carbon/zinc ratio was 2/1, the decrease of CO_2_ adsorption was observed as a consequence of the decrease of total pore volume, including micropores. 

Activation of the obtained materials with potassium oxalate increased the specific surface area values and CO_2_ adsorption values in comparison with the non-activated samples. 

As mentioned above, the activated samples modified using zinc nitrate exhibited the highest values of surface area and total pore volume in comparison with reference material RF7/1. For the material where the carbon-zinc ratio was 10/1, slightly higher values of CO_2_ adsorption were noticed than for the reference material: 6.79 mmol/g at 0 °C and 4.24 mmol/g at 25 °C for RF7/1 + Zn10/1 and 6.41 mmol/g at 0 °C and 4.13 mmol/g at 25 °C for RF7/1. Despite the increase of surface area and total pore volume for the sample RF7/1 + Zn5/1, lower values of CO_2_ adsorption were noticed, possibly due to an increase of volume of mesopores and a decrease in the volume of ultramicropores which, according to available works [20,47,48], are also responsible for the CO_2_ adsorption process. 

**Figure 12:**
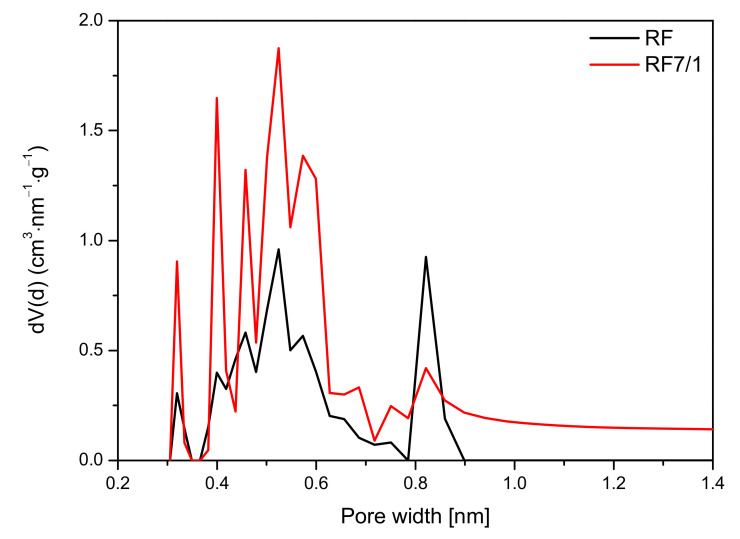
PSD of the nonactivated and activated sample.

The analysis of the micropore distribution in the reference materials showed that ultramicropores with diameters smaller than 1 nm were predominant. The diagram presented in Figure 12 indicates that the contribution of pores with a size below 1 nm was higher for an activated sample and amounted to 0.37 cm^3^/g than for a non-activated sample, for which this value was only 0.19 cm^3^/g. Undoubtedly, activation using potassium oxalate had a positive effect on the increasing contribution of ultamicropores responsible for the sorption of CO_2_. Due to this structure, the activated sample demonstrated greater adsorption capacity than was was reported in previous studies [8,9,19,29,30].

**Figure 13:**
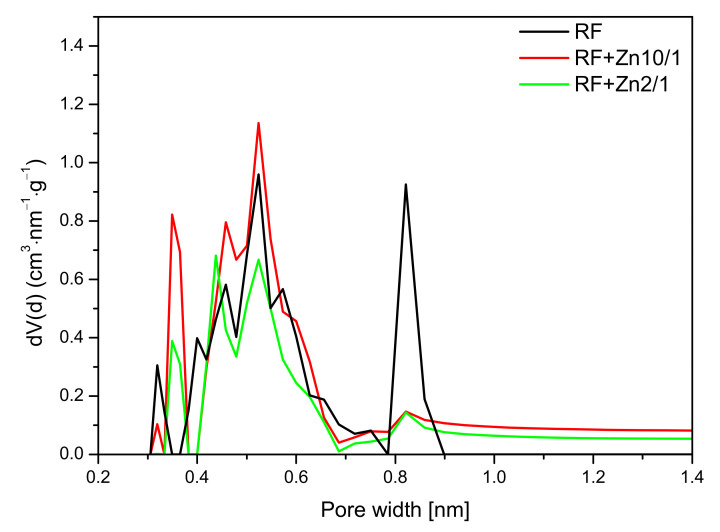
PSD of the non-activated samples modified with zinc nitrate.

For the nonactivated materials modified with Zn(NO_3_)_2_ in the ratio of 10/1 and 5/1, a slight increase in the CO_2_ adsorption at 0 °C in comparison with reference material (RF) was noticed. The pore size distributions of the samples with the highest (RF + Zn2/1) and the lowest (RF + Zn10/1) zinc content are presented in Figure 13. 

For the reference material RF, three groups of narrow micropores could be distinguished. The first group was 0.32 nm in size, but due to the size of CO_2_ molecules, the mentioned pores should not be used for CO_2_ adsorption [49]. For RF + Zn10/1, the contribution of the same pores was lower. However, pores of 0.35 nm size appeared. Moreover, for the material RF + Zn10/1, development of the pores in size of 0.55 nm was noticed. This could result in an increase in CO_2_ adsorption values. What is interesting is that the modified sample expressed a much lower contribution of pores of 0.85 nm in size, but it did not affect CO_2_ adsorption negatively. It seems that the higher content of pores below 0.7 nm was more important for CO_2_ adsorption, in consideration of previous studies [44,50]. 

For the sample characterized with the lowest CO_2_ adsorption capacity (RF + Zn2/1), the pore contribution in the range up to 0.7 nm was smaller than for the RF + Zn10/1 sample. It is worth noting that the contribution of the pores of 0.8 nm was similar for both materials (RF + Zn2/1, RF + Zn10/1) and did not affect the CO_2_ adsorption capacity, which confirms our previous observations.

**Figure 14:**
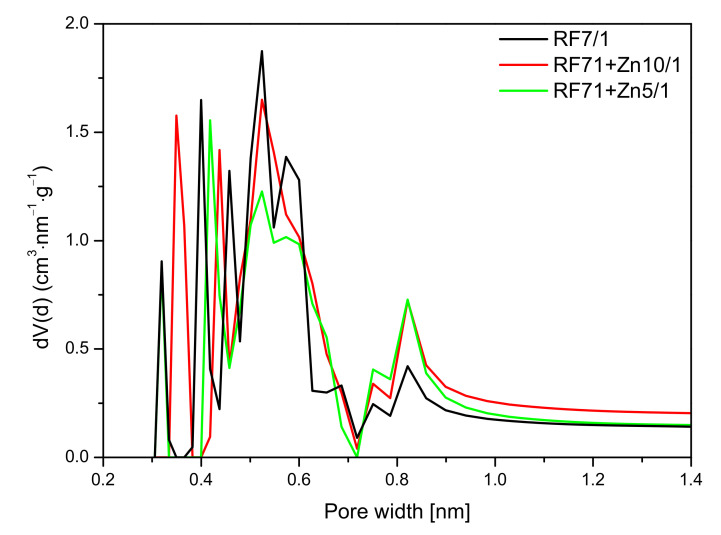
PSD of the activated samples modified with zinc nitrate.

A similar effect was observed in Figure 14, where the pore size distribution (PSD) of the activated and zinc nitrate-modified samples is shown. As mentioned before, the values of specific surface area and total pore volume calculated for the sample RF7/1 + Zn5/1 were larger than in the case of the reference material or even the material modified zinc nitrate RF7/1 + Zn10/1. Nevertheless, lower values of CO_2_ adsorption capacity were observed for this material. This was due to the decreased micropore volume in this sample, in favor of mesopores. In addition, it can be seen that, in the RF7/1 + Zn5/1 material, there were pores with a size below 0.35 nm which were not capable adsorbing CO_2_ (the kinetic diameter of the CO_2_ molecule is 0.33 nm). Similarly, as in the case of the reference material, for RF7/1 + Zn5/1, pores of a diameter of 0.35 nm were not observed, while the contribution of the pores in the range of 0.40–0.65 nm was lower than in the RF7/1 material. 

For the RF7/1 + Zn10/1 sample, a slight increase in CO_2_ adsorption compared to the RF7/1 reference material was noticed, probably due to the higher contribution of pores of 0.35 nm in diameter, which were not observed in the RF7/1 reference material.

**Figure 15:**
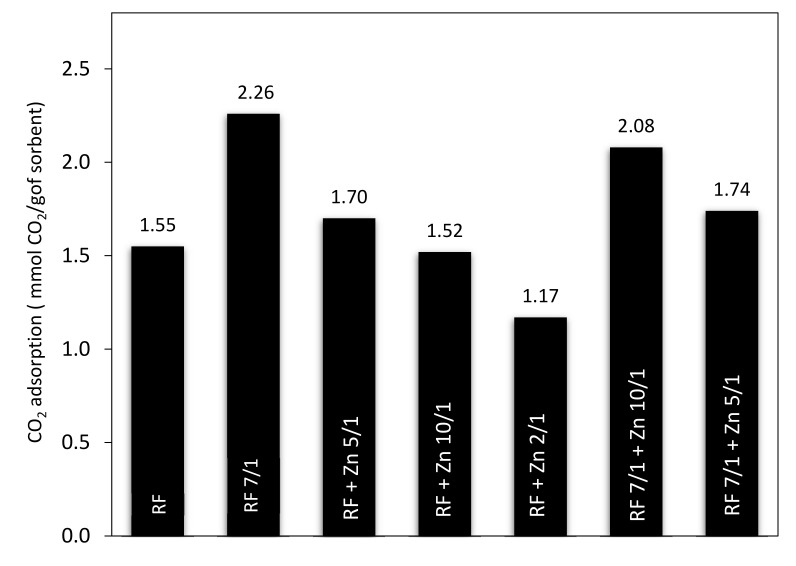
A comparison of CO_2_ adsorption capacity of nonactivated and activated materials modified with different amounts of zinc nitrate.

**Figure 16:**
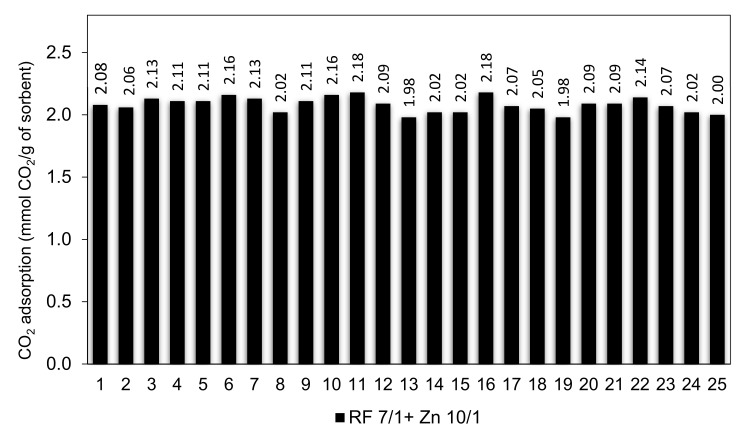
Multicyclic stability of CO_2_ adsorption-desorption of the RF 7/1+ Zn 10/1 sample.

Additional results of the CO_2_ adsorption experiments conducted on thermobalance at 40 °C for the unmodified materials and the carbons modified with different amounts of zinc nitrate as ZnO precursors, both nonactivated and activated with potassium oxalate, are shown in Figure 15 and Figure 16. The unmodified material (RF) and the sample after activation (RF 7/1) exhibited a CO_2_ adsorption capacity of 1.55 and 2.26 mmol/g, respectively. This corresponds to a 31.4% increase compared to the material before activation.

The sorption capacity changed after the modification of the carbon spheres with different amounts of zinc nitrate. The CO_2_ sorption capacities for RF + Zn5/1 and RF + Zn10/1 were 1.70 and 1.52 mmol/g, respectively, and reached the lowest value for the RF + Zn 2/1 (1.17 mmol/g). The ability of the materials to capture carbon dioxide decreased with the increase of the carbon-to-zinc ratio. As we can clearly see, the group of carbon spheres modified with zinc nitrate and activated with potassium oxalate achieved higher CO_2_ adsorption capacity than the samples prepared without activation. The CO_2_ sorption capacity for RF7/1 + Zn 5/1 and RF7/1 + Zn 10/1 reached 1.74 and 2.08 mmol/g, respectively. This corresponds to a 11.4 and 22.3% increase compared to the samples before activation. As mentioned in the characterization section, the activation process resulted in a significant increase in both surface area and micropore volume. As consequence, the CO_2_ adsorption capacity of the materials was higher than that of the non-activated ones. The observed differences in the adsorption capacity of the materials can be caused by various textural parameters. It is commonly known that not only the specific surface area [51] but also the structural parameters and porosity [47,52] play a very important role in enhancing the adsorption ability of the adsorbent.

Efficient CO_2_ adsorbents should not only possess high adsorption capacity but also should exhibit stable cyclic adsorption–desorption performance during prolonged operation.

The RF 7/1 + Zn 10/1 sample was selected to study the cyclic adsorption/desorption behavior because it had the largest adsorption capacity among all of the sorbents in this study. The cyclic CO_2_ adsorption capacities for 25 consecutive runs are shown in Figure 16.

The results show that the CO_2_ sorption capacity of 2.08 mmol/g and 2.06 mmol/g were achieved in the 1^st^ and 2^nd^ cycle. It was expected because CO_2_ molecules could get into all pores (especially ultramicropores) in the 1^st^ cycle of adsorption. After 2^nd^ cycle, the adsorption capacity was virtually stable and reached above 2.10 mmol/g between the 3^rd^ and 10^th^ cycles. The CO_2_ adsorption capacity of 2.18 mmol/g was reached after the 10^th^ and 16^th^ cycles. This corresponds to a 4.6% increase compared to the performance in the first cycle. The CO_2_ adsorption capacity slowly decreased to 2.0 mmol/g after 25 cycles. On the basis of the conducted research, we can conclude that the RF 7/1 + Zn 10/1 adsorbent shows excellent cyclability as well as very good regeneration capability, with a performance decay of less than 10% over up to 25 adsorption–desorption cycles.

## 4. Conclusions

The CO_2_ uptake of carbon spheres modified with zinc nitrate was presented. CO_2_ adsorption capacity measured at 0°C and 25 °C for non-activated samples doped with ZnO was higher than or similar to the reference material. The decrease of CO_2_ adsorption capacity took place only for the material with the carbon:zinc weight ratio of 2:1. The activation process using potassium oxalate allowed us to achieve higher values of CO_2_ adsorption than in the case of non-activated materials, but a higher zinc concentration in the samples resulted in a decrease of CO_2_ adsorption as well. It was confirmed that the specific surface area of the material was not a determining factor of the eadsorption properties, but pores smaller than 0.8 nm played a major role in this process. The highest CO_2_ adsorption capacity, measured at 40°C utilizing STA 449 C thermobalance, reaching 2.08 mmol/g, was observed in RF7/1 + Zn10/1. The calculated result is consistent with those obtained for CO_2_ adsorption carried out at 0 and 25°C. Furthermore, that sample not only displayed high CO_2_ adsorption capacity but also exhibited very good regeneration and stable performance during 25 consecutive adsorption–desorption cycles.

## Figures and Tables

**Table 1:** Textural parameters and CO_2_ sorption capacities for the obtained materials.

	S_BET_	TPV	V_s_ (<1 nm)	V_m_ (<2 nm)	V_meso_	CO_2_ 0 °C	CO_2_ 25 °C
	[m^2^/g]	[cm^3^/g]	[cm^3^/g]	[cm^3^/g]	[cm^3^/g]	[mmol/g]	[mmol/g]
RF	455	0.26	0.19	0.22	0.04	3.25	2.43
RF + Zn10/1	524	0.47	0.21	0.25	0.22	3.75	2.13
RF + Zn5/1	487	0.45	0.21	0.25	0.20	3.69	2.48
RF + Zn2/1	342	0.28	0.14	0.16	0.12	2.44	1.67
RF7/1	954	0.53	0.37	0.44	0.07	6.41	4.13
RF7/1 + Zn10/1	1152	0.63	0.38	0.53	0.10	6.79	4.24
RF7/1 + Zn5/1	1268	0.71	0.35	0.57	0.14	5.68	3.49

S_BET_—specific surface area; TPV—total pore volume; Vs—volume of ultramicropores with a diameter smaller than 1 nm; V_m_—volume of micropores with a diameter smaller than 2 nm; V_meso_—volume of mesopores with diameter from 2 to 50 nm.

## Data Availability

The data presented in this study are available on request from the corresponding author.
